# Effect of PAF on polyrnorphonuclear leucocyte plasma membrane polarity: a fluorescence study

**DOI:** 10.1155/S0962935193000225

**Published:** 1993

**Authors:** A. Kantar, P. L. Giorgi, R. Fiorini

**Affiliations:** 1 Departments of Pediatrics University of Ancona via Corridoni 11 Ancona 60123 Italy; 2 Departments of Biochemistry University of Ancona via Corridoni 11 Ancona 60123 Italy

## Abstract

The effect of PAF on the plasma membrane polarity of polymorphonuclear leukocytes (PMNs) was investigated by measuring the steady-state fluorescence emission spectra of 2-dimethylamino(6-1auroyl) naphthalene (Laurdan), which is known to be incorporated at the hydrophobic-hydrophilic interface of the bilayer, displaying spectral sensitivity to the polarity of its surrounding. Laurdan shows a marked steady-state emission blue-shift in non-polar solvents, with respect to polar solvents. Our results demonstrate that PAF (10^−7^ M) induces a blue shift of the fluorescence emission spectra of Laurdan. These changes are blocked in the presence of the PAF antagonist, L-659,989. Our data indicate that the interaction between PAF and PMNs is accompanied by a decrease in polarity in the hydrophobic-hydrophilic interface of the plasma membrane.


Mediators of Inflammation, 2, 135–141 (1993)

### NPC 15669 blocks neutrophil CD18 increase and lung injury during cardiopulmonary bypass in pigs

J. M. Bator,[1,CA] A. M. Gillinov,[2] K. J. Zehr,[2] J. M. Redmond,[2] I. C. Wilson,[2] A. Herskowitz,[3] J. R. Connor,[1] R. M. Burch,[1] D. E. Cameron[2]

[1]Scios Nova Inc., 6200 Freeport Centre, Baltimore, MD 21224, USA; [2]Johns Hopkins Medical Institution, Department of Cardiac Surgery, Baltimore, MD, USA; [3]Johns Hopkins Medical Institution, Division of Cardiology, Dept. of Medicine, Baltimore, MD, USA

[CA] Corresponding Author

**DURING cardiopulmonary bypass (CPB), neutrophils become activated due to contact with extracorporeal surfaces and binding of complement fragments C3a and C5a, leading to extravasation and subsequent tissue damage. In this study, the effects of the leumedin NPC 15669 (N - [9*H* - (2,7 - dimethylfluorenyl - 9 - methoxy) car - bonyl]-L-leucine), a leukocyte recruitment inhibitor, were evaluated in a pig model of CPB. NPC 15669 caused significant inhibition of CPB associated increase in CD18 upregulation, lung tissue myeloperoxidase content, and percentage wet weight compared to controls. Lung histology revealed clear airways and minimal neutrophil infiltration in treated animals *vs.* significant oedema and cellular infiltration in controls. It is concluded that CPB causes a dramatic increase in neutrophil CD18, and that leumedins are effective in inhibiting neutrophil activation and subsequent tissue injury when administered during CPB.**

**Key words:** Adhesion, Cardiopulmonary bypass, CD18, Leumedins, Neutrophils

### Introduction

Exposure of blood to extracorporeal artificial surfaces during procedures such as haemodialysis or cardiopulmonary bypass (CPB) has been shown to cause complement activation, resulting in stimulation of neutrophils.[1–5] During CPB, neutrophils become sequestered in tissues where they generate oxygen and hydroxyl free radicals and release granular constituents such as elastase and myeloperoxidase (MPO). The effects, seen most acutely in the lung, can include oedema, tissue damage, and organ dysfunction. Neutrophil activation during CPB has been demonstrated by measuring production of oxygen free radicals[6] and $LTB_4$,[7] release of lactoferrin,[8,9] elastase[8–10] and myeloperoxidase,[8,11] and increased formyl-methionyl-leucyl-phenylalanine (FMLP) receptor expression.[5]

Although the majority of patients recover satisfactorily from CPB, there are few effective means to prevent CPB associated inflammation and organ injury. Depleting the blood of neutrophils using leukocyte filters has shown some beneficial effect in a dog model of CPB.[12] Administration of steroids can attenuate some effects of complement mediated neutrophil activation, including neutropenia and chemotaxis, although complement activation itself may remain unchanged.[13] In humans, the use of non-steroidal anti-inflammatory drugs (NSAIDs) in the setting of CPB is undesirable because of their properties as anti-coagulants.

*In vitro*, activation of neutrophils by a variety of chemotactic factors, including C5a, $LTB_4$, and FMLP, has been shown to increase the level of expression of adhesion molecules, mainly the CD11b/CD18 heterodimer known as Mac-1. Mac-1 is now thought to be the principal molecule responsible for neutrophil adherence to endothelium.[14–17] *In vivo*, increased Mac-1 expression has been demonstrated in neutrophils of patients undergoing haemodialysis,[1] a procedure in which the blood is also exposed to artificial surfaces extracorporeally. Recently a new anti-inflammatory compound, leumedin NPC 15669, has shown efficacy *in vivo* in several neutrophil mediated inflammatory responses.[18,19] *In vitro*, NPC 15669 prevented the adherence of activated human neutrophils to cultured endothelial cells, and also inhibited upregulation of CD11b and CD18, the $\alpha$ and $\beta$ subunits of Mac-1.[20] It was felt that similar results might be obtained in an animal model of CPB. Preliminary evidence suggests that NPC 15669 may improve pulmonary function during CPB by inhibiting changes in arterial oxygen tension and pulmonary vascular resistance.[21] The present study was undertaken in pigs to determine whether (1) CD18, the common $\beta$ subunit of neutrophil Mac-1, was up-regulated during CPB; and (2) administration of NPC 15669 during CPB could affect CD18 expression and reduce neutrophil

© 1993 Rapid Communications of Oxford Ltd infiltration and subsequent tissue damage in the lungs.

### Materials and Methods

*Surgical manipulation:* Piglets weighing 10–12 kg were premedicated with ketamine (40 mg/kg, i.m.) and anaesthetized with Nembutal® (30 mg/kg, i.v.). A tracheostomy was performed and the pigs ventilated with 100% oxygen at a tidal volume of 15 ml/kg using a volume-cycled ventilator. PEEP was set at 5 cm $H_2O$ and anaesthesia was maintained with halothane, 0.5–1.0%. A polyethylene cannula was placed in the femoral artery for pressure monitoring and a Swan–Ganz catheter was passed through the external jugular vein. After median sternotomy, a polyethylene cannula was placed in the left atrium for pressure monitoring and blood sampling. All animals received heparin (300 U/kg, i.v.). Treated animals ($n = 5$) were given a loading dose of 10 mg/kg NPC 15669, i.v., over 10 min just before initiating CPB, followed by a constant infusion at 6 mg/kg/h. This infusion protocol resulted in a sustained plasma concentration of about 30 μg/ml (Fig. 1). Control animals ($n = 5$) received equal volumes of vehicle. CPB was initiated via cannulae placed in the right atrium and ascending aorta. A pulmonary artery vent was placed after initiation of bypass. The bypass circuit included a Sarns roller pump, a Bentley-10 Plus bubble oxygenator and a 40 μm in-line arterial filter. The prime consisted of 1200 ml of a mixture of fresh homologous blood and crystalloid in a proportion calculated to produce a final haematocrit of 25%. Animals were cooled to 28°C. Mean systemic arterial pressure was maintained at 50–55 mmHg with pump flows of 80 ml/kg when the temperature was greater than 32°C, and 50 ml/kg when the temperature was less than 32°C. Animals were warmed to 37°C during the final 30 min of CPB. At the conclusion of 2 h of CPB, an isoproterenol infusion (0.1 μg/kg/min) was started and the animals were weaned from bypass. The animals were stabilized over the next 30 min, and isoproterenol was discontinued. Animals were followed for 2 h after CPB. At the conclusion of this period, fully anaesthetized animals were sacrificed by intravenous injection of KCl. Sections of the lungs were removed for assessment of myeloperoxidase content and histological evaluation.

FIG. 1. Plasma concentration of NPC 15669 in pigs during and after CPB. Animals ($n = 5$) were given a loading dose of 10 mg/kg over 10 min just before the start of CPB, followed by a constant infusion at 6 mg/kg/h. A stable plasma concentration of approximately 30 μg/ml was achieved using this protocol.

All animals received humane care in compliance with the *Guide for the Care and Use of Laboratory Animals* published by the National Institutes of Health.

*Antibodies:* Antibody to porcine CD18 was the generous gift of Dr James E. K. Hildreth of Johns Hopkins Hospital.[22] Antibodies were produced from a murine hybridoma cell line, and were in the form of cell culture supernatant. Fluorescein-conjugated goat anti-mouse immunoglobulin (FITC-GAMIg) was purchased from Sigma.

*Measurement of neutrophil CD18:* For *in vitro* measurements, venous blood was obtained from a heparinized normal pig. Equal volumes (100 μl each) of whole blood and Hepes buffered Hank's balanced salt solution without calcium or magnesium ($HHBSS^-$) with or without NPC 15669 (final concentration 100 μM), were incubated at room temperature for 15 min. Appropriate samples were treated with complement component C5a (Sigma), final concentration 0.5–20 nM, diluted in buffer containing calcium (HHBSS) at 37°C for 20 min. Samples were then placed on ice and incubated with anti-porcine CD18 antibody (undiluted, 100 μl) followed by labelling with FITC-GAMIg. After washing, erythrocytes were lysed and leukocytes fixed using the Coulter Whole Blood Lysing Reagent Kit (Coulter Electronics Inc., Hialeah, FL, USA). Flow cytometric analysis of cellular fluorescence was performed on a Coulter Profile II. Neutrophils were gated on the basis of forward and 90° light scatter, and fluorescence was measured on a linear scale. Fluorescence values were converted to percentage increase over control, and expressed as mean ±S.E.M.

For measurements during CPB, arterial blood samples were obtained at the following time points: pre-sternotomy, post-sternotomy, 15, 60, 90, and

120 min after initiation of CPB, and 30, 60, 90 and 120 min post-CPB. Duplicate 100 $\mu$l samples were added to polypropylene tubes on ice and 100 $\mu$l anti-porcine CD18 antibody was added. Samples were further processed as detailed above. Flow cytometric analysis of cellular fluorescence was performed similarly.

*Measurement of plasma NPC 15669 concentration:* At the time points indicated above, arterial blood was collected and centrifuged at 3 000 × $g$ for 15 min, and the plasma removed. Plasma samples were stored at −70°C until ready for use. An equal volume of ice-cold ethanol was added to the plasma, vortex mixed, and then incubated on ice for 10 min. Each tube was vortex mixed again and centrifuged for 2 min in a microcentrifuge, then 100 $\mu$l was injected onto a Regis SPS-C8 HPLC column, 25 cm × 4.6 mm. The ultraviolet absorbing plasma components elute near the solvent front and the compound is retained and elutes later. An HP 1040 with diode array detector was used; mobile phase was 55% acetonitrile in 0.1% TFA, at a flow rate of 1 ml/min. Detection was at 265 nm. Using this system, retention time of the internal standard was 11 min, and NPC 15669, 13 min. Standard curves of NPC 15669 were constructed using spiked plasma samples.

*Measurement of lung tissue myeloperoxidase:* Lung biopsies (200 mg) were placed into 2 ml 0.5% hexyldecyltrimethylammonium bromide in 50 mM potassium phosphate, pH 6.0. Tissues were then homogenized using a Polytron (Brinkmann) for 30 s at a setting of 6.5. The tissue was then sonicated using a Kontes Ultrasonic Cell Disrupter with a 3 mm probe. Finally, the tissue was put through three freeze (dry ice/methanol)–thaw (37°C water bath) cycles. The disrupted tissue was then centrifuged at 42 000 × $g$ for 15 min at 4°C. Aliquots (0.1 ml) of the supernatant were added to 2.9 ml assay buffer (0.167 mg/ml *o*-dianisidine, 0.05% hydrogen peroxide, 50 $\mu$M potassium phosphate, pH 6.0). Absorbance at 460 nm was measured after a 2 min incubation and converted to $\mu$mol/10 mg tissue using an extinction coefficient of $1.13 \times 10^4$ for oxidized dianisidine.

*Histology:* Perfusion fixed samples of lung tissue from five control and five treated animals were processed for haematoxylin and eosin staining and histologic examination. Samples from each lobe of the left lung (total five samples per lung, ten fields each) were examined under low (25 ×) power.

*Determination of lung tissue percentage wet weights:* After sacrifice, a 10–20 mg section of the right lung was removed. The tissue was weighed and then incubated at 100°C for 24 h, after which it was weighed again. Percentage lung wet weight was calculated as follows:

percentage wet weight

= (wet weight − dry weight)/wet weight × 100

*Plasma neutrophil counts:* Arterial blood samples for determination of complete blood counts were drawn at the indicated times. Complete blood counts were performed by Coulter Counter and differential cell counts were done manually. Counts are expressed as mean ± S.E.M. ($n = 5$).

*Statistical analysis:* All values are expressed as mean ± S.E.M. Significance was determined using Student's *t*-test. Comparison between groups was made using ANOVA (analysis of variance for repeated measures).

### Results

*Inhibition of CD18 upregulation* in vitro*:* Because neutrophil activation during CPB is associated with complement activation, the C5a mediated increase was measured in CD18 on porcine neutrophils *in vitro*, and the ability of NPC 15669 to inhibit this increase was assessed. The results are shown in Fig. 2. Over the given concentration range, human C5a caused an increase in CD18 expression approximately 40% over control. In previous studies,[20] it was found that 100 $\mu$M NPC 15669 caused nearly

FIG. 2. NPC 15669 inhibition of C5a mediated increase in CD18 expression *in vitro*. Buffer (100 $\mu$l HHBSS$^-$) with (○) or without (●) NPC 15669 (final concentration 100 $\mu$M) was added to 100 $\mu$l of heparinized blood from a normal pig. After a 15 min incubation at room temperature, one-tenth volume HHBSS with or without C5a (final concentration 0.5–20 nM) was added. After 20 min at 37°C, samples were placed on ice and anti-porcine CD18 antibody or HHBSS was added to appropriate samples. All samples were then labelled with FITC-GAMIg. Red blood cells were lysed and leukocytes fixed prior to measurement of cellular fluorescence. Fluorescence values were converted to percentage increase over control and expressed as the mean ± S.E.M. ($n = 3$). *$p < 0.05$.

complete inhibition of stimulated neutrophil adherence to endothelial cells *in vitro* ($IC_{50} \approx 30\ \mu M$). Therefore, NPC 15669 at this concentration was tested for its ability to inhibit C5a mediated CD18 upregulation. As shown in Fig. 2, pretreatment of cells with 100 μM NPC 15669 resulted in CD18 expression of just 20% above baseline. In the range of 1–10 nM C5a, this inhibition was statistically significant ($p < 0.05$). A similar inhibition of CD18 expression has been observed when stimulated human neutrophils are pretreated with equivalent concentrations of NPC 15669.[20]

At this concentration of NPC 15669, neutrophils are >99% viable, as determined by LDH release and trypan blue exclusion. Furthermore, the absence of toxic effects of NPC 15669 *in vitro* has been demonstrated in wash-out experiments. When neutrophils are incubated with 100 μM NPC 15669 for 20 min and then washed free of drug, cellular activation, as demonstrated by homotypic aggregation and adherence to endothelium, can be induced by a variety of chemotactic factors (data not shown).

*CD18 expression on neutrophils during CPB:* The expression of CD18, the β subunit of the Mac-1 adhesion molecule, on neutrophils of five pigs receiving NPC 15669 and five pigs receiving vehicle alone was measured. The results are displayed in Fig. 3. In pigs receiving vehicle, CD18 expression

FIG. 3. NPC 15669 inhibition of increase in CD18 expression on neutrophils during CPB. Five piglets received NPC 15669 just prior to and during CPB (○, 10 mg/kg bolus followed by 6 mg/kg/h i.v.), and five received vehicle alone (●). Arterial blood samples were obtained at time points indicated. Anti-porcine CD18 antibody or buffer was added to 100 μl aliquots of whole blood and incubated on ice for 30 min. All samples were then labelled with FITC-GAMIg. Red cells were lysed and leukocytes fixed prior to measurement of cellular fluorescence. Fluorescence was measured on a linear scale. Values were converted to percentage increase over baseline and expressed as mean ± S.E.M. ($n = 5$ for each group). CD18 expression in the treated group was significantly less than that in the untreated group ($p < 0.02$, ANOVA for repeated measures).

began to increase after sternotomy and continued to rise throughout bypass to a level greater than 150% over baseline. CD18 expression remained elevated for the duration of the study (2 h post-bypass). In pigs treated with NPC 15669, however, CD18 expression was significantly less than in untreated controls. While CD18 expression in control animals continued to rise during the 2 h of bypass, that from leumedin treated animals plateaued at just 30–50% above baseline during the same time period. CD18 expression on leumedin treated pigs increased somewhat after bypass; however, expression was still significantly lower than in untreated controls (ANOVA, $p < 0.02$).

*Myeloperoxidase levels in lung tissue:* Myeloperoxidase (MPO) is released from primary (azurophilic) granules of activated neutrophils. Measurement of tissue MPO content therefore allows an accurate assessment of the degree of neutrophil infiltration.[23] Myeloperoxidase was measured in lung tissue sections taken after each animal was sacrificed. In normal pigs, lung tissue MPO was determined to be 16 ± 3 μmol/10 mg tissue ($n = 3$). In untreated pigs that underwent CPB, tissue MPO increased more than six-fold to 106 ± 9 μmol/10 mg tissue. In pigs treated with NPC 15669, however, lung tissue MPO increased only three-fold to 48 ± 4 μmol/mg tissue ($p = 0.0004$, $n = 5$ in each group). These results imply that neutrophil infiltration into the lungs was significantly inhibited in pigs given NPC 15669 during bypass.

*Lung tissue percentage wet weights:* The percentage wet weight reflects the relative fluid content of the lung tissue. Thus, an increase in percentage wet weight indicates oedema. The wet weight of normal lung tissue was determined to be about 84%. In untreated animals, this value increased to 88.7 ± 0.4% 2 h post-bypass. In animals pre-treated with NPC 15669, however, the percentage wet weight increased to just 85.4 ± 0.8, a value significantly less than in untreated controls ($p < 0.03$, $n = 5$ in each group). These results indicate that NPC 15669 prevented the CPB-associated lung oedema observed in untreated animals.

*Lung histology:* Histological observation of lung tissue biopsies revealed a striking difference between treated and untreated animals. Sections of lung from each animal in both groups were examined. Representative sections are shown in Fig. 4(a) and (b). In animals given NPC 15669, pulmonary structure was virtually normal with little or no oedema and few sequestered neutrophils. The alveolar septae are thin and the alveolar spaces appear clear (Fig. 4a). In contrast, lungs from untreated animals revealed multi-focal areas of pulmonary oedema, including interstitial and

FIG. 4. (a) Photomicrograph of a representative lung section taken from a pig given NPC 15669 during CPB. The normal lung architecture is intact. The alveolar septae are thin and the alveolar spaces appear empty (haematoxylin and eosin, magnification 25×); (b) Photomicrograph of a representative lung section taken from a non-treated animal. The normal alveolar architecture is obscured. The alveolar spaces are filled with oedema fluid and individual alveolar spaces cannot be identified (haematoxylin and eosin, 25×).

FIG. 5. Plasma neutrophil counts from control (●) and NPC 15669 treated (○) animals. Arterial blood samples for determination of complete blood counts were drawn at the indicated times. Neutrophil numbers were determined manually from blood smears. Counts are expressed as mean ± S.E.M. ($n = 5$).

intra-alveolar oedema and haemorrhage. In addition, clusters of red blood cells were evident in the alveolar spaces, and interstitial capillaries contained many clusters of neutrophils (Fig. 4b).

*Circulating neutrophil counts:* In both control and leumedin treated pigs, circulating neutrophil counts decreased from approximately 8 000/mm$^3$ pre-sternotomy to less than 500/mm$^3$ 90 min after initiation of CPB (Fig. 5). However, both lung MPO content and histological evaluation indicate that the number of neutrophils sequestered in the lung tissue was much greater in control than in treated animals. Thus, the low numbers of circulating neutrophils in the leumedin treated group may be a reflection of marginated, rather than extravasated neutrophils (see Discussion). Untreated animals remained neutropenic for the duration of the post-bypass observation period (2 h). In treated animals, however, neutrophil counts increased to over 2 000/mm$^3$ at 2 h post-bypass ($p = 0.06$), indicating a trend toward reversal of leukopenia in the treated group.

### Discussion

The *in vivo* anti-inflammatory activity of NPC 15669 has recently been reported. These results suggested that NPC 15669 might offer protection from neutrophil mediated lung injury during CPB. Therefore, the purposes of this study were (1) to demonstrate that neutrophil adhesion molecules were upregulated during CPB in a porcine model; and (2) to determine whether the anti-inflammatory compound NPC 15669 could inhibit neutrophil adhesion molecule upregulation in pigs during CPB, thereby reducing the neutrophil mediated tissue injury that often accompanies CPB. It was demonstrated that CD18 expression increases more than 150% during porcine CPB, and remains elevated up to 2 h post-CPB. When NPC 15669 was administered to pigs during CPB, inhibition of neutrophil CD18 upregulation was observed. In contrast to the increase of over 150% in control animals, less than 50% increase was observed in animals given NPC 15669. Post-CPB, CD18 expression in leumedin treated animals increased somewhat; however, it was still significantly less than that in control animals at all measured timepoints. Based on these results and those from previous *in vitro* neutrophil adherence assays, it is concluded that inhibition of CD18 expression contributed to a reduction of neutrophil infiltration into lung tissue.

Although two $\beta_2$ integrins, LFA-1 (CD11a/CD18) and Mac-1 (CD11b/CD18), contribute to neutrophil adhesion, only Mac-1 expression increases upon stimulation by chemotactic factors. Lo and coworkers[16] have shown that treatment of neutrophils with phorbol dibutyrate (PDB), tumour necrosis factor (TNF), or C5a caused little to no change in the expression of LFA-1. In contrast,

Mac-1 expression increased by 150%, 100%, and 50% upon stimulation with PDB, TNF, and C5a, respectively. While the expression of a third $\beta_2$ integrin, gp150,95 (CD11c/CD18), has been shown to increase upon stimulation, its contribution to neutrophil adhesion is relatively insignificant.[24,25] In the present experiments, use of an antibody to only the common $\beta$ subunit CD18 does not permit differentiation among LFA-1, Mac-1, and gp150,95 expression. However, previous studies indicated that NPC 15669 could completely block neutrophil adherence,[20] an event mediated by only LFA-1 and Mac-1. It is conceivable, therefore, that NPC 15669 affects all three $\beta_2$ integrins, either by inhibiting upregulation, or by inhibiting other as yet undefined qualitative alterations in the receptor.

In other animal models,[26–28] administration of monoclonal antibodies against CD18 has been shown to block neutrophil mediated reperfusion injury. Unfortunately, it was not possible to test anti-CD18 antibodies in this model of CPB because sufficient quantities of anti-porcine CD18 were not available. However, NPC 15669 has been compared to anti-integrin antibodies in a model of LPS mediated endotoxin challenge in mice.[29] In this model, untreated animals become irreversibly leukopenic, and death results from organ failure following neutrophil sequestration in the tissues. In mice given a lethal dose of LPS (50 mg/kg), NPC 15669 was comparable to anti-CD11b and CD18 antibodies in its ability to improve survival and reverse neutropenia.

Lung histology indicated that neutrophil sequestration was inhibited by NPC 15669. This was substantiated by measurement of lung tissue MPO content 2 h post-CPB. In leumedin treated animals, MPO content was reduced by more than 50% compared to controls, indicating significant inhibition of neutrophil infiltration.

The percentage wet weight in end-point lung biopsies was also determined. The results indicate that control pigs undergoing CPB develop considerable lung oedema. NPC 15669 administered to pigs during CPB largely prevented the oedema seen in control pigs. Histological evaluation of lung biopsies confirmed marked oedema in untreated pigs. In contrast, lung sections from animals receiving NPC 15669 showed unobstructed airways and the absence of oedema. It is clear that NPC 15669 exerted a marked effect in inhibiting the neutrophil accumulation, oedema, and subsequent tissue injury associated with CPB.

Neutropenia often occurs during CPB.[4,5] In the pig model described here, very few circulating neutrophils were present during CPB in both control and leumedin treated animals. Similarly, when leumedins were tested in a murine model of LPS induced leukopenia, both control and treated animals were initially neutropenic;[19] however, neutropenia was dose dependently reversed in leumedin treated animals over the course of about 24 h. Thus in the present study, the initial neutropenia, as well as the trend toward recovery from neutropenia during the 2 h post-bypass period, was consistent with earlier observations in the murine model. Since tissue MPO content in leumedin treated animals was significantly less than in untreated controls, the apparent neutropenia does not necessarily imply neutrophil extravasation. It is possible that during CPB, the majority of neutrophils were marginated, that is, slowly rolling or moderately adherent to endothelial cells lining the vessels. This loose adherence could be mediated by the LECAM-1 adhesion molecule, which is constitutively expressed,[30] rather than by Mac-1, which is upregulated upon neutrophil activation.

In summary, this study has demonstrated that neutrophil CD18 expression is upregulated during CPB. This, combined with other less well understood activation events, likely leads directly to increased neutrophil adherence to endothelium, infiltration into extravascular space, and tissue injury resulting from release of granular constituents and generation of oxygen derived free radicals. The novel anti-inflammatory compound NPC 15669, when given to pigs during CPB, offered significant protection from the damaging effects by inhibiting neutrophil activation. This inhibition was demonstrated by a reduction in neutrophil CD18 expression, lung tissue myeloperoxidase content, and percentage wet weight. Histological evidence confirmed a marked reduction in the numbers of infiltrating neutrophils in the lung. Leumedins may offer significant therapeutic potential in cardiopulmonary bypass and in other clinical situations where neutrophil mediated tissue injury is known to occur.

### References

1. Arnaout MA, Hakim RM, Todd RF III, Dana N, Colten HR. Increased expression of an adhesion-promoting surface glycoprotein in the granulocytopenia of hemodialysis. *N Engl J Med* 1985; **312**: 457–462.
2. Chenoweth DE, Cooper SW, Hugli TE, Stewart RW, Blackstone EH, Kirklin JW. Complement activation during cardiopulmonary bypass. Evidence for generation of C3a and C5a anaphylatoxins. *N Engl J Med* 1981; **304**: 497–503.
3. Howard RJ, Crain C, Franzini DA, Hood CI, Hugli TE. Effects of cardiopulmonary bypass on pulmonary leukostasis and complement activation. *Arch Surg* 1988; **123**: 1496–1501.
4. Hammerschmidt DE, Stroncek DF, Bowers TK, *et al.* Complement activation and neutropenia occurring during cardiopulmonary bypass. *J Thorac Cardiovasc Surg* 1981; **81**: 370–377.
5. Tennenberg SD, Clardy CW, Bailey WW, Solomkin JS. Complement activation and lung permeability during cardiopulmonary bypass. *Ann Thorac Surg* 1990; **50**: 597–601.
6. Cavarocchi NC, England MD, Schaff HV, *et al.* Oxygen free radical generation during cardiopulmonary bypass: correlation with complement activation. *Circulation* 1986; **74**(suppl. III): 130–133.
7. Utoh J, Yamamoto T, Kambara T, Goto H, Miyauchi Y. Perioperative alteration of neutrophil functions after open heart and other major surgeries. A comparative study. *J Cardiovasc Surg* 1989; **30**: 692–695.
8. Riegel W, Spillner G, Schlosser V, Hörl WH. Plasma levels of main granulocyte components during cardiopulmonary bypass. *J Thorac Cardiovasc Surg* 1988; **95**: 1014–1019.

9. Wachtfogel YT, Kucich U, Greenplate J, *et al.* Human neutrophil degranulation during extracorporeal circulation. *Blood* 1987; **69(1)**: 324–330.
10. Antonsen S, Brandslund I, Clemensen S, Sofeldt S, Madsen T, Alstrup P. Neutrophil lysosomal enzyme release and complement activation during cardiopulmonary bypass. *Scand J Thorac Cardiovasc Surg* 1987; **21**: 47–52.
11. Faymonville ME, Pincemail J, Duchateau J, *et al.* Myeloperoxidase and elastase as markers of leukocyte activation during cardiopulmonary bypass in humans. *J Thorac Cardiovasc Surg* 1991; **102**: 309–317.
12. Bando K, Pillai R, Cameron DE, *et al.* Leukocyte depletion ameliorates free radical-mediated lung injury after cardiopulmonary bypass. *J Cardiovasc Surg* 1990; **99**: 873–877.
13. Tennenberg SD, Bailey WW, Cotta LA, Brodt JK, Solomkin JS. The effects of methylprednisolone on complement-mediated neutrophil activation during cardiopulmonary bypass. *Surgery* 1986; **100**: 134–142.
14. Anderson DC, Miller LJ, Schmalstieg FC, Rothlein R, Springer TA. Contributions of the Mac-1 glycoprotein family to adherence-dependent granulocyte functions: structure–function assessments employing subunit-specific monoclonal antibodies. *J Immunol* 1986; **137**: 15–27.
15. Smith CW, Marlin SD, Rothlein R, Toman C, Anderson DC. Cooperative interactions of LFA-1 and MAC-1 with intercellular adhesion molecule-1 in facilitating adherence and transendothelial migration of human neutrophils *in vitro*. *J Clin Invest* 1989; **83**: 2008–2017.
16. Lo SK, Detmers PA, Levin SM, Wright SD. Transient adhesion of neutrophils to endothelium. *J Exp Med* 1989; **169**: 1779–1793.
17. Schleiffenbaum B, Moser R, Patarroyo M, Fehr J. The cell surface glycoprotein Mac-1 (CD11b/CD18) mediates neutrophil adhesion and modulates degranulation independently of its quantitative cell surface expression. *J Immunol* 1989; **142**: 3537–3545.
18. Burch RM, Weitzberg M, Blok N, *et al.* *N*-(fluorenyl-9-methoxy-carbonyl) amino acids, a class of anti-inflammatory agents with a different mechanism of action. 1988 *Proc Natl Acad Sci USA* 1991; **88**: 355–359.
19. Noronha-Blob L, Lowe VC, Weitzberg M, Burch RM. NPC 15669 enhances survival and reverses leukopenia in endotoxin-treated mice. *Eur J Pharmacol* 1991; **199**: 387–388.
20. Bator JM, Weitzberg M, Burch RM. *N*-[9H-(2,7-dimethylfluorenyl-9-methoxy)carbonyl]-L-leucine, NPC 15669, prevents neutrophil adherence to endothelium and inhibits CD11b/CD18 upregulation. *Immunopharmacol* 1992; **23**: 139–149.
21. Gillinov AM, Redmond JM, Zehr KJ, *et al.* Inhibition of neutrophil adhesion reduces pulmonary injury due to cardiopulmonary bypass. *Circulation* 1992; **86**(Suppl 1): 354.
22. Hildreth JEK, Holt V, August JT, Pescovitz MD. Monoclonal antibodies against porcine LFA-1: species cross-reactivity and functional effects of $\beta$-subunit-specific antibodies. *Mol Immunol* 1989; **26**: 883–895.
23. Grisham MB, Benoit JN, Granger DN. Assessment of leukocyte involvement during ischemia and reperfusion of intestine. *Methods Enzymol* 1990; **186**: 729–742.
24. Lo SK, van Seventer GA, Levin SM, Wright SD. Function and regulation of the neutrophil MEL-14 antigen *in vivo*: comparison with LFA-1 and Mac-1. *J Immunol* 1989; **143**: 3325–3329.
25. Springer TA, Miller LJ, Anderson DC. p150, 95, the third member of the Mac-1, LFA-1 human leukocyte adhesion glycoprotein family. *J Immunol* 1986; **136**: 240–245.
26. Horgan JM, Wright SD, Malik AB. Antibody against leukocyte integrin (CD18) prevents reperfusion-induced lung vascular injury. *Am J Physiol (Lung Cell Mol Physiol 3)*; **259**: L315–L319.
27. Jaeschke H, Farhood A, Smith CW. Neutrophils contribute to ischemia/reperfusion injury in rat liver *in vivo*. *FASEB J* 1990; **4**: 3355–3359.
28. Vedder NB, Winn RK, Rice CL, Chi EY, Arfors K-E, Harlan JM. Inhibition of leukocyte adherence by anti-CD18 monoclonal antibody attenuates reperfusion injury in the rabbit ear. *Proc Natl Acad Sci USA* 1990; **87**: 2643–2646.
29. Burch RM, Noronha-Blob L, Bator JM, Lowe VC, Sullivan JP. Mice treated with a leumedin or antibody to a $\beta_2$ integrin to inhibit leukocyte sequestration survive endotoxin challenge. *J Immunol* 1993; in press.
30. von Andrian UH, Chambears JD, McEvoy LM, Bargatze RF, Arfors K-E, Butcher EC. Two-step model of leukocyte-endothelial cell interaction in inflammation: distinct roles for LECAM-1 and the leukocyte $\beta_2$ integrins *in vivo*. *Proc Natl Acad Sci USA* 1991; **88**: 7538–7542.

ACKNOWLEDGEMENTS. This work was supported in part by USPHS NIH Grant No. RO1-HL47191-01. The authors thank Cheryl Sowards for excellent preparation of the manuscript, and Melissa Haggerty and Ernest King for technical assistance with surgery.

**Received 28 December 1992; accepted in revised form 3 February 1993**
